# Strengthening a Medium-Carbon Low-Alloy Steel by Nanosized Grains: The Role of Asymmetrical Rolling

**DOI:** 10.3390/nano13050956

**Published:** 2023-03-06

**Authors:** Youzhi Li, Yongfeng Shen, Sixin Zhao, Weina Zhang, Wenying Xue

**Affiliations:** 1 Key Laboratory for Anisotropy and Texture of Materials (Ministry of Education), School of Materials Science and Engineering, Northeastern University, Shenyang 110819, China; 2 Central Research Institute, BaoShan Iron & Steel Co., Ltd., Shanghai 201999, China; 3 The State Key Lab of Rolling & Automation, Northeastern University, Shenyang 110819, China

**Keywords:** medium-carbon steel, asymmetrical rolling, gradient structure, ultrafine grain, nanoindentation

## Abstract

A medium-carbon low-alloy steel was prepared via the asymmetric rolling process with different ratios of upper and down roll velocities. Subsequently, the microstructure and mechanical properties were explored by using SEM, EBSD, TEM, tensile tests and nanoindentation. The results show that asymmetrical rolling (ASR) can significantly improve strength while retaining good ductility compared with conventional symmetrical rolling. The yield strength and tensile strength of the ASR-steel are 1292 ± 10 MPa and 1357 ± 10 MPa, respectively, which are higher than the values of 1113 ± 10 MPa and 1185 ± 10 MPa for the SR-steel. The ASR-steel retains good ductility of 16.5 ± 0.5%. The significant increase in strength is related to the joint actions of the ultrafine grains, dense dislocations and a large number of nanosized precipitates. This is mainly because of the introduction of extra shear stress on the edge under asymmetric rolling, which induces gradient structural changes hence increasing the density of geometrically necessary dislocations.

## 1. Introduction

The medium-carbon low-alloy steel with chemical compositions of Fe-0.4C-0.09V-1.05Cr-1.01Mo-0.73Mn-0.61Ni-0.17Si in weight percentage (wt.%) is widely used in various industrial fields including the production of train axles, reactor pressure vessels, oil pipelines and so on [1,2,3]. The key advantage is because of its excellent work-hardening ability and high yield strength; however, the industrial application of common medium-carbon low-alloy steel is limited because of its coarse grain and low yield strength. Therefore, improving the strength of medium-carbon low-alloy steel has become an important subject. Among the various strengthening mechanisms of metals, grain refinement is one of the most effective methods, which leads to less deterioration of plasticity. At present, severe plastic deformation (SPD) preparation technology has been widely used in the processing of ultrafine-grained metal materials. Ultrafine fine-grained materials can be prepared by obtaining large nucleation work through SPD. Since the beginning of the new century, there has been much research on the mass production of ultrafine-grained metals by SPD. Nanoscale ultrafine-grained metal materials (UFG) have been successfully prepared by SPD processes such as equal channel angular extrusion (ECPA), accumulative roll-bonding (ARB), high-pressure torsional deformation (HPT), high-speed friction welding (HSFW) [4], hydrostatic extrusion (HE) [5] and multi-directional forging (MF).

Previously, Jia et al. carried out innovative processes including the intercritical rolling and controllable annealing of medium-carbon low-alloy steel, leading to a microstructure consisting of a large number of nanoscales Fe_3_C precipitates in the UFG ferrite matrix [4]. The good combination of high-strength plasticity is mainly related to the dispersive distribution of nano-Fe_3_C particles in the UFG ferrite. The former improves the strength by delaying dislocation movement, while the latter reserves enough space for dislocation slip [6,7,8]. Subsequently, Liang et al. reported that the UFG ferritic steels had an advantage in tensile strength and elongation-to-failure (ε_f_) at 600 °C, especially at the strain rate of 0.0017 s^−1^, with high σ_UTS_ of 510 MPa and excellent low temperature (<0.42T_m_) and superplasticity (110% of total elongation) [9]. Wang et al. prepared ultrafine grains (UFG) with an average grain size of 980 nm by ECAP at room temperature for 16 consecutive passes and deep cold rolling with a 50% pressure vector, and obtained excellent mechanical properties. Through research, it is found that the high strength is mainly due to the joint action of ECAP and cryogenic rolling [10]. Jafarian et al. found that the yield strength and ultimate tensile strength of Fe-28.5Ni steel can be significantly improved after only the first stacking period, and ultrafine grains gradually form with the increase of the stacking period in the subsequent stacking process. Additionally, the strength first increased and then decreased, and the elongation decreased slightly [11]. Although the above-mentioned SPD methods can significantly refine grains, their application range is mostly soft metals or small-sized steel materials, which is not suitable for the development of ultrafine-grained steel materials, and the steel field is greatly limited. Therefore, research on the rolling method has become the top priority in the steel industry. Some scholars said that, when compared with SR, ASR has a higher grain refinement ability [12,13,14], and the ASR process is considered the most suitable method for large-scale production [15].

Shear deformation will be caused in the deformation zone of the metal rolled piece during ASR. An additional shear strain (γ) was given by Saito et al. [16].

(1) $$\phi =\sqrt{1+{\left\{\frac{{\left(1-r\right)}^{2}}{r(2-r)}\tan\theta \right\}}^{2}}$$

(2) $$\gamma =2\sqrt{\phi^{2}-1}\ln\frac{1}{1-r}$$

where r is the reduction ratio and θ is the shear angle after ASR, which can be added to the rolling reduction using uneven-sized rolls or an uneven roll speed during rolling. This additional shear strain could provide a shear texture in rolled strips and was reported to refine the grain size, providing a larger plastic strain compared to the SR in a lab-scale hot rolling.

Under the additional shear stress field, the deformation mode will be changed from a single compression deformation mode to a compression-shear deformation mode, which will excite more slip systems to participate in slip and cross slip in the deformation zone, resulting in the enhancement of rotational cubic texture, and the shear zone is concentrated with very high local plastic deformation and high deformation energy storage [17]. Therefore, ASR technology has many advantages, including reducing rolling pressure, improving pass reduction rate, improving machining efficiency and remarkable grain refinement. There are two main ways to conduct ASR: one is ASR with different ratios of upper and down roll velocities (RUDV); the other is ASR with different roll diameters [18]. Nevertheless, the previous studies on grain refinement via ASR were mainly performed in Mg, Al, Cu and high entropy alloys [12,19,20,21]. Most of the studies on ASR were carried out at room temperature and high temperature (480 °C) [22,23,24]. As far as the steel is concerned, ASR has not been applied between the Ac_1_–Ac_3_ transformation region (~750–820 °C). It is very challenging because the high temperature is apt to induce grain growth. Therefore, there is currently a gap in our understanding of the influence of asymmetric warm rolling on the microstructure evolution of medium-carbon low-alloy steel, thus affecting the grain gradient change. As mentioned earlier, the grain refinement behavior of medium-carbon low-alloy steel by rolling has always been the subject of research, and some results have been achieved [6,8,25,26].

In this study, a medium-carbon low-alloy steel with chemical compositions of Fe-0.4C-0.09V-1.05Cr-1.01Mo-0.78Mn in weight percentage (wt.%), was processed by an innovative process including hot rolling incorporated with subsequent warm rolling. The improved combination of strength and plasticity is achieved by controlling RUDV, and the reason for strength change is explored, which provides guidance for further exploring the application of ASR in the field of advanced high-strength steel.

## 2. Experimental Procedure

### 2.1. Material and Methods

In this study, an ingot of medium-carbon low-alloy steel was melted in a vacuum induction furnace, and its chemical compositions were determined by atomic emission spectrometry (AES), as shown in Table 1. It should be pointed out that the Ni content of the steel is much lower (0.007 wt.%) than that of the traditional 45CrNiMoV steel (1.0 wt.%), which is cost-effective. The ingot was processed into a blank that was hot rolled to a slab with dimensions of 100 mm × 100 mm × 120 mm. Finally, the resultant slab was heated by using a resistance furnace at 1200 °C and kept for 2 h to remove the inhomogeneous microstructure. After 9 passes of continuous hot rolling at 1050 °C, the obtained plate thickness of 6 mm was air-cooled to room temperature.

**Figure 1:**
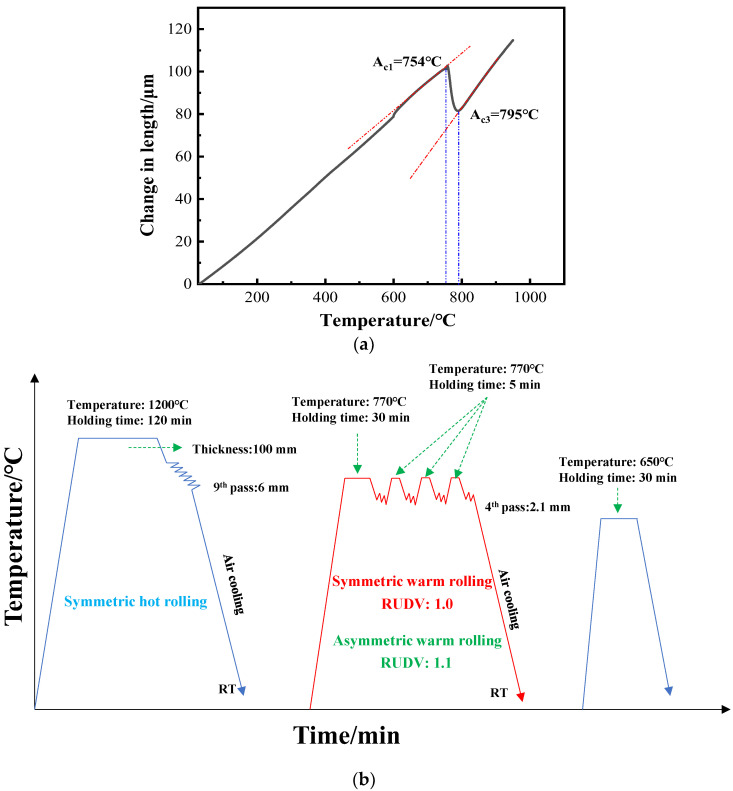
(**a**) Thermal expansion curve for measuring the transformation points of a Fe-0.4C-0.09V-1.05Cr-1.01Mo-0.78Mn steel and (**b**) a schematic illustration shows the joint processes of hot rolling and asymmetric warm rolling for preparing the UFG steel.

To determine the phase transition temperatures, a cylindrical thermal expansion sample with a size of φ 4 mm × 10 mm from the center of the hot rolled plate using a wire-cut EDM machine. Subsequently, the sample was cleaned by using an ultrasonic cleaning machine. The expansion test was conducted on a DIL805 A/D thermal dilatometer (Germany, Ochtrup, Baehr-Thermo Ltd.) and the processes include: (i) heating the sample to 600 °C at a rate of 10 °C/s, (ii) heating the sample to 900 °C at 0.1 °C/s and holding it for 180 s, (iii) cooling the sample to 25 °C at a rate of 10 °C/s. The results show that the austenite-start temperature (Ac_1_) and -finish temperature (Ac_3_) are 754 °C and 795 °C, respectively (Figure 1a). To study the effect of ASR on the microstructure and mechanical properties of the steel, the thickness of the steel was reduced from 100 mm to 6 mm after 9 passes of hot rolling at 1200 °C, and then air-cooled to room temperature. Subsequently, the hot rolled plate with a thickness of 6 mm was heated to 770 °C (Ac_1_~Ac_3_) and kept for 30 min, and then rolled at a different speed according to the RUDV of 1.1:1 (ASR) and 1:1 (SR). The plate was reheated at 770 °C for 5 min in a resistant furnace during the interval between the neighboring passes (Figure 1b). 

**Figure 2:**
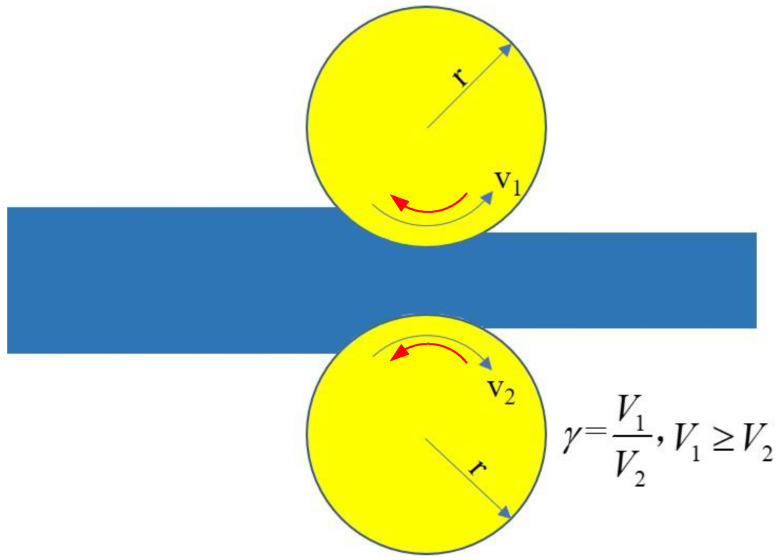
Schematic diagram of asymmetric rolling (V1: circular velocity of upper work roll, V2: circular velocity of bottom work roll).

Considering repeatability, the even-numbered rolling passes must be adopted, and the rolling direction between the two adjacent passes must be changed (Figure 2). The change of rolling direction is mainly based on the requirements of uniformity and multi-directivity, which promotes the start of different slip systems. Increasing passes is beneficial for dislocation movement between different slip systems, thus increasing dislocation density and obtaining gradient structure. In this study, the plate was rolled four passes and then air-cooled to room temperature. Finally, the resultant plate with a thickness of 2.1 mm was annealed at 650 °C for 30 min, and the obtained steels were named the SR-steel and ASR-steel, respectively.

### 2.2. Uniaxial Tensile Tests

To measure mechanical properties, uniaxial tensile tests were carried out on a Shimadzu AG-X plus pc-controlled mechanical testing system (Shimadzu Co. Ltd., Kyoto, Japan). The strain rate was 0.017 s^−1^ and the tests were performed at 25 °C. The dog-bone-shaped specimens were cut from the plate and annealed at 650 °C for 30 min, along the rolling direction (RD) by using an electron discharge machine. The tested specimens had the gage dimensions of 6 mm × 3 mm × 1.5 mm. To guarantee the test accuracy, the specimens were sequentially polished to get rid of the surface defects which may induce stress concentration during tensile tests, by using sandpapers with the different grades of 800#, 1000#, 1200# and 1500#.

### 2.3. Microstructure Characterization

Scanning electron microscope (SEM), electron back-scattered diffraction (EBSD), transmission electron microscopy (TEM) and X-ray diffraction (XRD) were used to characterize the microstructure of the steels prepared by using ASR and SR. To explore the microstructural evolution of steels under different rolling methods, the samples were cut and then mechanically polished and finally electro-polished for obtaining a high level of recognition during EBSD measurement. The electrolyte consisted of 10 vol.% HClO_4_ and 90 vol.% C_2_H_5_OH. The EBSD analysis was conducted on a JSM-6510A SEM by using a scan step size of 50 nm and a voltage of 20 kV. A scanned region of 10 × 2 mm^2^ was large enough for obtaining statistically representative results which contained the microstructure information in detail. Subsequently, the obtained data were evaluated by using orientation imaging microscopy (OIM, HKL-Channel 5) software. To explore the distribution of geometrically necessary dislocations (GNDs) and the variation trend of the GNDs density with strain rates, the corresponding kernel average misorientation (KAM) maps were given. The limit of the KAM misorientation was defined as 5°. For the TEM observations, the discs with a diameter of 3 mm were cut from the as-prepared steel and the deformed simple; then, the discs were mechanically polished to a thickness of ~50 μm and subsequently thinned in an electrolyte containing 8 vol.% perchlorate and 92 vol.% ethanol at −10 °C with dry ice, by using a twin-jet electrochemical polishing operated at 27 V. TEM observations were carried out in a Tecnai G2 F20 microscope at 200 kV. The average size and volume fraction of precipitates were measured by using Image Pro Plus 6.0 software, by counting the particles from 12 pictures obtained from TEM.

### 2.4. Nanoindentation Tests

To characterize the effect of ASR on the microstructure and mechanical properties of the medium-carbon low-alloy steel, the micromechanical properties of the edge and center of the samples were measured via nanoindentation. Since the indentations were significantly affected by the loading rate [27], all measurements were carried out to a maximum load of 3000 μN at a constant loading rate of 50 μN/and then held for 5 s. The same process was repeated to reach different depths of the prepared samples along the RD-ND plane. Subsequently, it is completely unloaded within 5 s. Finally, 24 indentations were obtained on the edge and center of the sample. Due to the shear stress at the edge, the edge grains were different from the others. To reduce the detection error, the indentations were carried out near the edge and the center following different matrixes. The matrixes at the edge and center were 2 × 6 and 3 × 4, respectively, and the distance between the two points was 5 μm. 

## 3. Results and Discussion

### 3.1. Microstructure Characterized via SEM Observations

**Figure 3:**
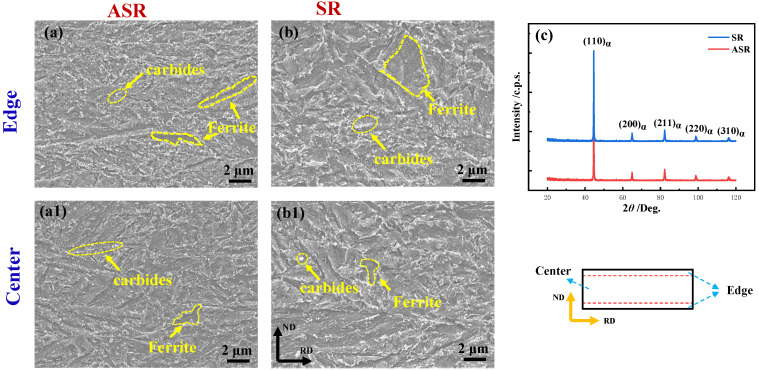
SEM pictures of the ASR-steel (**a**,**a1**), SR-steel (**b**,**b1**) and the corresponding XRD patterns (**c**).

Figure 3 shows the microstructure of the as-prepared steel along the rolling direction (RD) and normal direction (ND). A similar characteristic between ASR-steel and SR-steel is that their microstructure consists of ferrite and carbide. The microstructure in dark is ferrite while that in white is carbide, most of which distribute at the grain boundaries and the others uniformly locate in the grain. In addition, one can see that the ferritic grains mainly appear in strips, and the key reason is related to the rolling process. The ferritic grains were elongated into long strips during the warm rolling; thus, the deformed grains were preserved in the following tempering process. However, the grain deformation degree is intensified because of the extra shear stress introduced in the ASR process, leading to the grain sizes in ASR-steel (Figure 3a,a1) being less than those in the SR-steel (Figure 3b,b1). Moreover, no significant difference can be seen between the edge and the center, i.e., either the morphology and size of the grains together with those of precipitates are approximately identical. The formation of numerous carbides can be associated with the preparation processing. When the steels were held between Ac_1_–Ac_3_, the microstructure consisted of ferrite and austenite. During warm rolling, the spherical carbides precipitated at the grain boundaries of austenite. In the subsequent cooling process, austenite transformed to ferrite while the carbides shifted with the movement of grain boundaries. Thus, a large number of carbides were scattered in the ferritic matrix. Figure 3c shows the XRD patterns of ASR- and SR-steel, indicating that the two plates of steel consist of the single bcc phase. 

### 3.2. Grain Size and Dislocation Density Measured by EBSD

**Figure 4:**
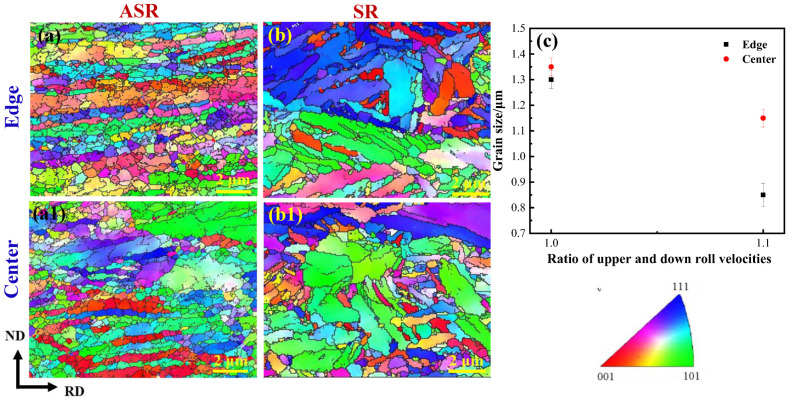
EBSD inverse-pole-figure (IPF) microstructure maps exhibit the morphology of grains in ASR-steel (**a**,**a1**) and SR-steel (**b**,**b1**) and the statistical grain sizes (**c**). Grains with {001}, {111} and {101} orientations parallel to the rolling direction are marked in red, blue and green, respectively.

Figure 4 shows the EBSD inverse-pole-figure (IPF) microstructure maps that exhibit the morphology of grains in the edge and center of the ASR- and SR-steel. The main common feature between the two steels is that numerous grains are elongated along the rolling direction (RD). Nevertheless, it is interesting to notice that the misorientation of grains in the two steels is different. The misorientation of those in the ASR-steel is approximately parallel with RD (Figure 4a,a1); however, the grains in the SR-steel have an angle of ~45° with RD (Figure 4b,b1). The key reason is that, under the same rolling reduction, the greater the shear stress, the greater the shear angle [Equations (1) and (2)]. The shear stress of ASR is greater than SR, which effectively promotes the rotation of grains during the rolling, thus pushing the grains to parallel with RD. Another distinct difference between the two steels is that the grain size of the ASR-steel is significantly smaller than that of the SR-steel. The average grain size is 850 nm at the edge of the ASR-steel (Figure 4a) while that is 1300 nm for the SR-steel (Figure 4b). At the center, the average grain sizes are 1150 nm (Figure 4a1) and 1350 nm (Figure 4b1) for the two steels, respectively. In addition, based on the statistical results, the average thickness of grains is 510 nm at the edge while that is 760 nm in the center for the ASR-steel. The aspect ratio of grains at the edge and in the center of the ASR-steel is about 3:7 and 1:2, respectively. By contrast, the aspect ratio of grain at the edge and in the center of the SR-steel is about 4:5 and 3:5, respectively. The results show that ASR is more effective for grain refinement compared to SR and that the grains at the edge are more effectively refined in comparison with those at the center. This is mainly because the shear deformation at the edge is severer during ASR, and the multi-directional strains render geometric sufficient conditions for grain fragmentation, introducing a higher number of grain boundaries after ASR than SR. 

**Figure 5:**
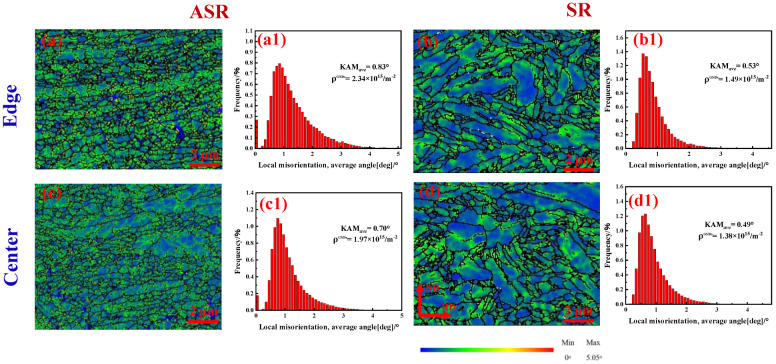
Kernel average misorientation (KAM) maps and statistic histograms obtained from the KAM maps. (**a**,**a1,c**,**c1**) ASR; (**b**,**b1,d**,**d1**) SR.

Figure 5 shows the KAM pictures of the edge and center of the ASR- and SR-steel, and the corresponding average KAM values can be calculated by using the equation:

(3) $${\mathrm{KAM}}_{ave}=\exp\left[\frac{1}{N}\sum_{l}^{i}\ln{\mathrm{KAM}}_{L,i}\right]$$

where KAM*_L,i_* is the local KAM value at point *i*, *N* is the number of points in the measured area and KAM*_ave_* is the average misorientation angle. Consequently, the average KAM values of SR-steel are 0.53° and 0.49° at the edge in the center, respectively. In contrast, the average KAM values of ASR-steel are significantly higher than those of SR-steel, with values of 0.83° and 0.70° at the edge and in the center. 

In addition, the KAM pictures show the difference in geometrically necessary dislocations (GNDs) density between ASR- and SR-steel (Figure 5a–d). The KAM values around grain boundaries are higher than those in other regions. This means that a large number of dislocations accumulate around grain boundaries; hence, grain boundaries are considered an effective obstacle to dislocation movement, which has been proved in early reports [6,7]. On the other hand, the average KAM values of the ASR-steel (Figure 5a,a1,c,c1) are higher than those of the SR-steel (Figure 5b,b1,d,d1). Moreover, at the same rolling process, the average KAM values at the edge (Figure 5a,b) are higher than those in the center (Figure 5c,d).

On the other hand, the higher KAM value near the grain boundary indicates that high-density dislocations accumulate around the grain boundary caused by severe deformation. Generally, the high KAM value is related to the high GNDs density produced during deformation. The GNDs density can be estimated by the following formula [28,29]:

(4) $$\rho^{GNDs}=\frac{2{\mathrm{KAM}}_{ave}}{\mu b}$$

where *μ* is the selected scanning step of EBSD measurement (50 nm) and *b* is the Burgers vector with a value of 0.248 nm. Consequently, the calculated density of GNDs at the edge and in the center of the ASR-steel are 2.34 × 10^15^ m^−2^ and 1.97 × 10^15^ m^−2^, respectively, which are significantly higher than the values of 1.49 × 10^15^ m^−2^ and 1.38 × 10^15^ m^−2^ at the corresponding regions in the SR-steel. The results suggest that ASR affects not only the grain size (Figure 4) but also dislocation density.

**Figure 6:**
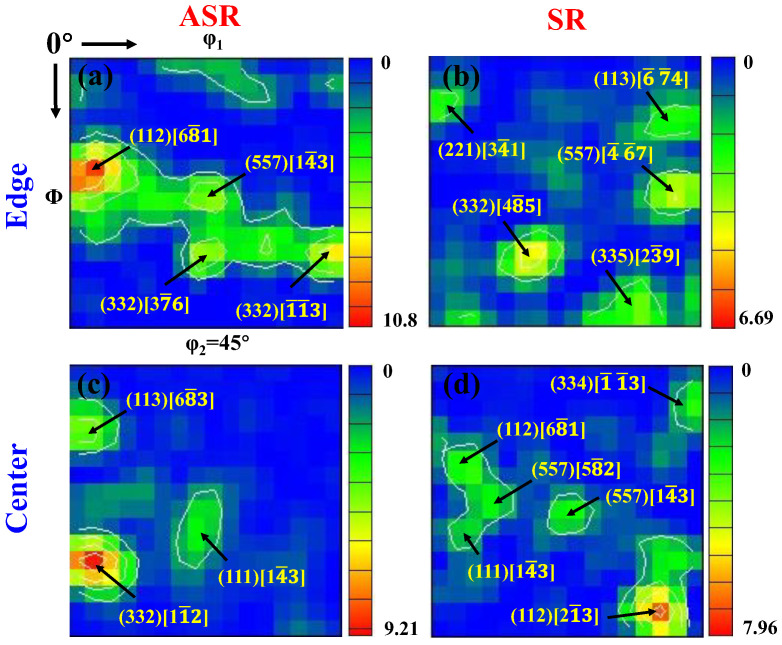
Orientation distribution function (ODF) at φ_2_ = 45° sections of the ASR- and SR-steel: (**a**) edge of the ASR-steel, (**b**) edge of the SR-steel, (**c**) center of the ASR-steel and (**d**) center of the SR-steel.

Figure 6 shows the ODF pictures at the edge and in the center of the ASR- and SR-steel. For the ASR-steel, the texture intensity at the edge and in the center is stronger (edge: 10.8, center: 9.21) than those for the SR-steel (edge: 6.69, center: 7.96). The deformation textures of the SR-steel are mainly (332)[4$\bar{8}5$] and (112)[2$\bar{1}3$] components. However, the deformation textures of the ASR-steel are dominated by the (112)[6$\bar{8}1$] and (332)[1$\bar{1}2$] components. The results show that the deformation texture is very strong in both the ASR- and SR-steel. Compared with the SR-steel, the textures at the edge and in the center are distinctly stronger in the ASR-steel. The increase in texture intensity means that the equivalent strain change of the rolled steel is strong, indicating that the dislocation density is very high, which is well consistent with the KAM diagram (Figure 5).

### 3.3. TEM Observations of the Microstructure

**Figure 7:**
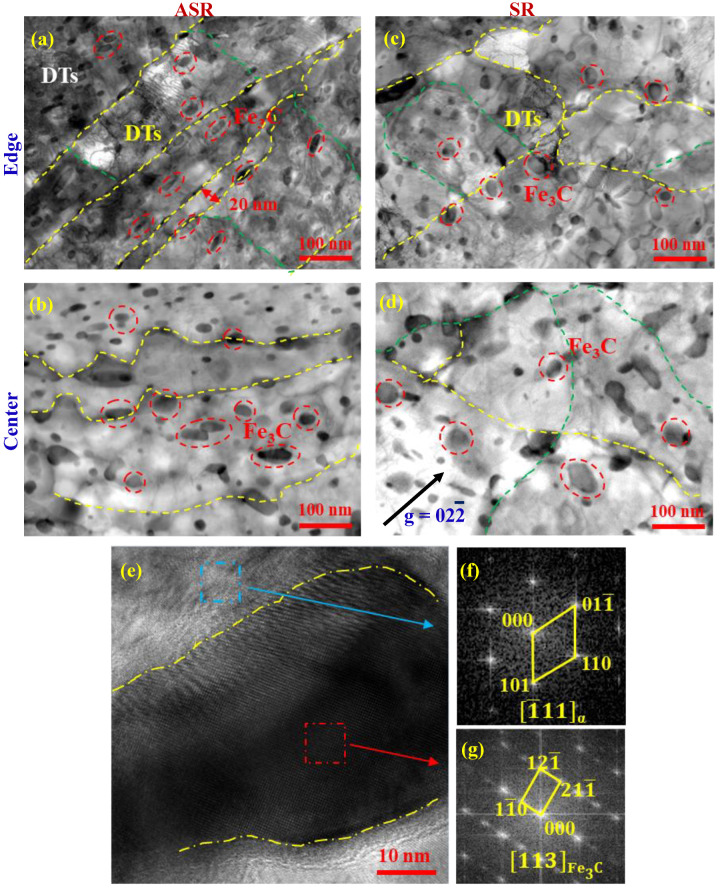
TEM pictures show the morphologies of the ASR-steel at the edge (**a**) and in the center (**b**), as well as those of the SR-steel at the edge (**c**) and in the center (**d**), obtained with g = 02$\bar{2}$. High-resolution image (**e**) and the corresponding Fast-Fourier-Transition (FFT) diffraction patterns of the matrix (**f**) and the precipitate (**g**) in the ASR-steel. DTs: dense dislocation tangle.

Figure 7 shows the TEM images of the microstructures at the edge and in the center of the ASR- and SR-steel. The grain boundaries are indicated by yellow dot lines and those of subgrains are revealed with blue dot lines. It can be seen that many grains are elongated due to ASR, which well matches the SEM and EBSD observations (Figure 3 and Figure 4). Many elongated grains have thicknesses of several hundreds of nanometers; however, the thickness of an elongated grain is as fine as 20 nm (Figure 7a). It should be pointed out that the lengths of the grains are too large to fully see (Figure 7a,b), which are approximately several microns tested via EBSD (Figure 4). In addition, the subgrains have a size of several hundreds of nanometers which is far below the results obtained from SEM and EBSD, due to the limited resolution. Another distinct characteristic is that there are a large number of spherical precipitates in the matrixes of the ASR- and SR-steel, either at the edges (Figure 7a,c) or in the centers (Figure 7b,d). It is worth mentioning that the majority of precipitates in the SR-steel have an equiaxed-like morphology. However, many precipitates in the ASR-steel exist in ellipses deflected along the direction parallel to grain boundaries (Figure 7a,b). The reason may be related to the shear stress caused by ASR. The most impressive feature is that the precipitates occupy about the half area of the matrix in the ASR-steel. Nano-measure software was used to measure the size of the precipitated phase at different regions, and the average sizes of the particles in the ASR-steel are 24 nm and 22 nm at the edge and in the center, while those are 32 nm and 37 nm, respectively, in the SR-steel. Hence, the average size of the precipitates is ~20 nm in the ASR-steel (Figure 7a) while that is 32 nm in the SR-steel (Figure 7c). In addition, dislocation tangles (DTs) can be observed at the edges of the ASR- and SR-steel (Figure 7a,c). Dense dislocations are always companied by the precipitates, suggesting that the nanosized particles can effectively hinder dislocation movements. In contrast, dislocation density in the centers of the ASR- and SR-steel is very low (Figure 7b,d), due to the low deformation degree. High-resolution transmission electron microscopy (HRTEM) ((Figure 7e) and the corresponding Fast-Fourier-Transition (FFT) patterns (Figure 7f,g) show that the matrix has a body-cubic-center (bcc, ferrite) and the precipitate is Fe_3_C.

### 3.4. Mechanical Properties under Tensile Tests

**Figure 8:**
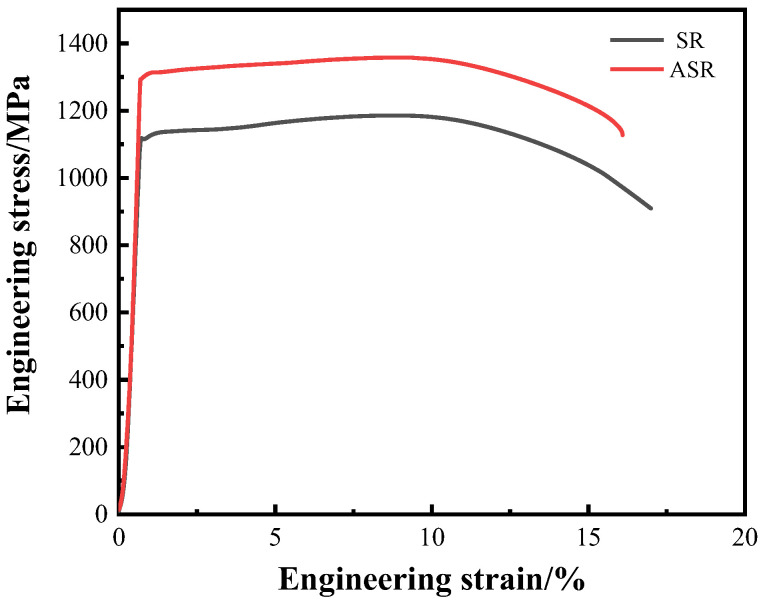
Engineering stress-strain curves of the ASR- and SR-steel.

Figure 8 shows the engineering stress-strain curves of the ASR- and SR-steel. The tensile properties of the medium-carbon low-alloy steel are closely associated with the change of RUDV. The yield strength (at 0.2 offsets) and tensile strength of the ASR-steel are 1292 ± 10 MPa and 1357 ± 10 MPa, respectively, which are distinctly higher than the values of 1113 ± 10 MPa and 1185 ± 10 MPa for the SR-steel. Moreover, the strengths of the steels in this study have great improvement in comparison with the reported comparts [30,31]. For example, the yield strength and tensile strength of a medium-carbon ultrafine-grained ferritic steel with spherical cementite were only 425 MPa and 645 MPa [30], respectively, and those of a medium-carbon low-alloy steel are 870 MPa and 930 MPa after the spheroidizing treatment [31]. SR and ASR have the advantage to obtain high strength. Nevertheless, it is worth noting that the yield strength and tensile strength of ASR-steel are higher than those of the SR-steel. During ASR, a large number of dislocations are retained in the shear band, which increases the resistance of dislocation movements during the subsequent tensile test. Consequently, ASR-steel has high strength. Meanwhile, the increase in dislocation density promotes the local strain concentration which provides a crack source in the ASR-steel during the tensile test. Hence, the ductility of the ASR-steel is 16.5 ± 0.5%, which slightly decreases (~1%) in comparison with the SR-steel.

In addition, the difference in mechanical properties between the ASR- and SR-steel can also be attributed to the different stresses applied to the edges and centers of the ASR-steel: Compared with the SR-steel, the ASR-steel suffered not only compressive stress but also increasing shear stress, i.e., the stress state is different; thus, the deformation mode of the rolled piece has changed during the rolling process. During ASR, the rolled plate first underwent shear deformation, and then shear deformation happened. Consequently, the ASR-steel bears not only strain $\varepsilon_{x}$ and $\varepsilon_{z}$, but also shear strain $\varepsilon_{xz}$ in comparison with the SR-steel. When only the planar deformation is considered, the equivalent strain of materials can be calculated by using the following equation:

(5) $$\varepsilon_{eq}=\sqrt{\frac{2}{9}}\sqrt{{\left(\varepsilon_{x}-\varepsilon_{y}\right)}^{2}+{\left(\varepsilon_{y}-\varepsilon_{z}\right)}^{2}+{\left(\varepsilon_{z}-\varepsilon_{x}\right)}^{2}+6\left(\varepsilon_{xy}{}^{2}+\varepsilon_{yz}{}^{2}+\varepsilon_{xz}{}^{2}\right)}$$

As a result, it can be seen that the equivalent strain exerted by ASR is greater than that from SR when the rolled plate bears the identical reduction. Thus, the deformation degree of the ASR-steel is larger than that of the SR-steel, and the grain refinement degree is significantly obvious. Additionally, because of the greater stress on the edge, the grains on the edge are finer; hence, the strength contribution is accordingly higher.

### 3.5. Micromechanical Properties under Nanoindentation

**Figure 9:**
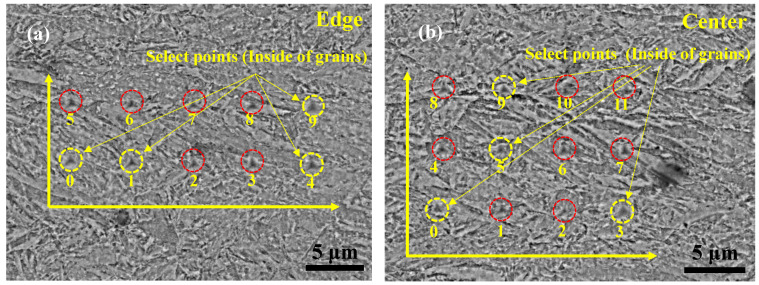
SEM observations show the morphologies and positions of nanoindentations at the edge (**a**) and in the center (**b**) of the ASR-steel. Red and yellow circles indicate the indentations at the grain boundaries and in the grains, respectively.

EBSD and TEM observations show that the grain refinement at the edge is better than that in the center of the ASR-steel. Therefore, nanoindentations were carried out on the regions near the edge (100 μm away from the surface) and in the center (1000 μm away from the surface) of the specimens, respectively. Figure 9 exhibits the distributions of indents at the edge (Figure 9a) and in the center (Figure 9b). One can discern that some indentations locate in the interior of the grains while others situate at the grain boundaries, which are indicated by yellow and red circles, respectively. In particular, most of the intragranular indentations were shown as equilateral triangles with large depth while those at grain boundaries mostly exhibit the form of isosceles triangles with small depth. Indeed, previous studies suggested that the hardness near the grain boundary region increases because of the obstruction of the grain boundary to dislocation movements [32]. Meanwhile, the plastic deformation induced by indentation can cause the work hardening of the neighboring regions; hence, the indentation depth at the grain boundary is quite different due to the loading sequences [33]. To explore the influence of grain gradient on elastic modulus and hardness, the intragranular indentations at the edge (0, 1, 4 and 9, Figure 9a) and in the center (0, 3, 5 and 9, Figure 9b) were selected. The corresponding contact depth, hardness and elastic modulus of the indentations are listed in Table 2. One can see that the indentations in the center usually have large contact depth (*h_c_*) while those at the edge have small *h_c_*.

Based on the following equations, the hardness and elastic modulus of indentation points in the yellow circle at the edge and in the center are counted. The obtained results are listed in Table 2.

(6) $$E_{r}=\frac{\sqrt{\pi }}{2\beta }\times \frac{S}{\sqrt{A}}$$

where *β* is the indenter shape factor— the quadrangular pyramid indenter is used in this experiment—and the *β* value of 1.012. *S = dP/dh* is obtained by taking the slope of the unloaded linear portion of the nanoindentation curve. *A* is the contact area of nanoindentation.

(7) $$A=24.56h_{c}^{2}+C_{0}h_{c}+{\sum_{i=1}^{4}C_{i}(\sqrt{h_{c}})}^{i}$$

where *h_c_* is the depth of contact of the indenter, and for an ideal indenter *C_0_* = *C_i_* = 0, so the contact area A = 24.56$h_{c}^{2}$.

(8) $$H=\frac{P_{\max}}{A}$$

where *H* is the hardness and *P_max_* is the maximum load in μN. *A* is the contact area of nanoindentation.

**Figure 10:**
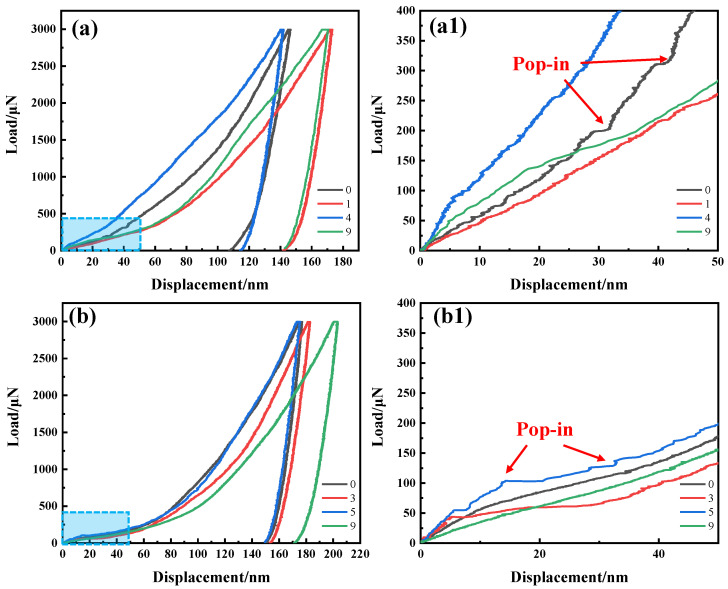
Loading-displacement curves of nanoindentations of the ASR steel: indentation at the edge (**a**) and the magnification of the curves within the rectangle (**a1**), indentation in the center (**b**) and the corresponding magnification of the curves within the rectangle (**b1**).

Figure 10 exhibits the load-displacement curves of the intragranular indentations at the edge (Figure 10a,a1) and in the center (Figure 10b,b1) of the ASR-steel. Despite the identical loading and loading rate, the displacement of each indenter is different. By referring to Table 2, one can see that the 0# indenter at the edge has the smallest *h_c_* which is 107 nm, while the 9# indenter at the center has the largest *h_c_* of 171 nm. In general, the *h_c_* of the indenters at the edge is significantly smaller than that of the indenters in the center. Moreover, it can be seen from the loading-unloading curves (Figure 10a,b) that with the increase of loading force, the slope of the curve tends to be flat at first and rises sharply at last. By magnifying the sections in the rectangles (Figure 10a,a1,b,b1), it is found that the displacements are different at the same loading; moreover, the larger the displacement the higher the hardness. The key reason is related to grain refinement strengthening. For example, at the identical indentation depth of 40 nm, the loading force is as high as 315 μN at the edge (0# in Figure 10a1) while that is only 121 μN in the center (9# in Figure 10b1). Upon the loading, the local work hardening occurs because of the grain refinement strengthening. Thus, a greater force is needed when the material is pressed into the same depth. 

In particular, some discontinuous serrated steps appear in the loading stage of the curves (Figure 10a1,b1), which is usually called serrated rheological behavior or pop-in phenomenon [34]. The sawtooth behavior is generally attributed to the nucleation of dislocations and the dynamic strain aging caused by the interaction between diffused solute atoms and moving dislocations [34]. Here it is related to the pinning/unpinning of moving dislocations in displacement-controlled compression experiments [35,36]. During the loading process, when the nanoindenter causes deformation of the material edge, dislocations nucleate and start to move. However, when dislocation encounters obstacles such as dislocation and precipitation, it is temporarily stopped. Meanwhile, movable interstitial elements such as carbon rapidly diffuse to low-energy positions, further strengthening the barriers to dislocations [35]. Thus, the movement of dislocations is further limited by the existence of solute atoms. As the stress to make the tip displacement increases, these dislocations helped to overcome obstacles and slip continually. Subsequently, they encounter new obstacles and are pinned again, and the process is repeated [34].

**Figure 11:**
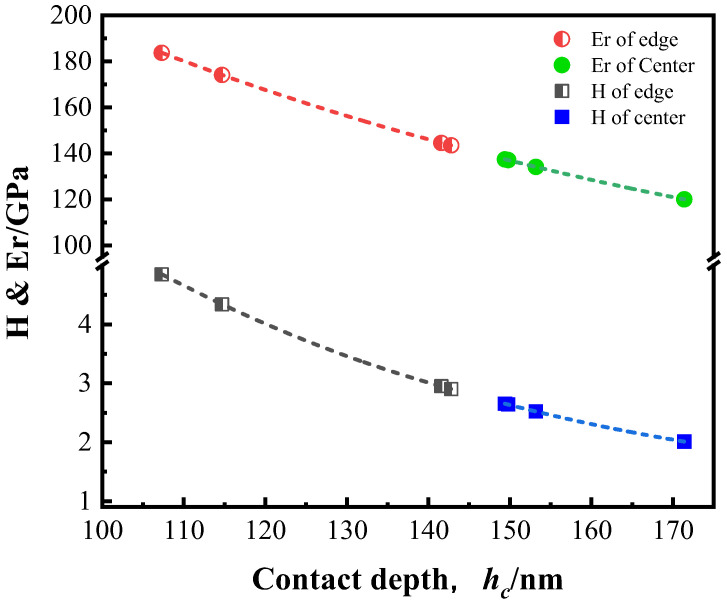
Evolutions of hardness and elastic modulus with increasing contact depth ($h_{c}$) in the center and at the edge of the ASR-steel.

Figure 11 shows the variation of *Er* and *H* with *h_c_* in ASR at a constant loading rate of 50 μN/s and a maximum load of 3000 μN. Obviously, *Er* and *H* decrease with the increase of *h_c_*, either at the edge or in the center. Nevertheless, *Er* and *H* of the edge are higher than those of the center. By fitting the experimental data, the following equations are obtained:

(9) $$Er_{edge}{=50+390e}^{-h_{c}/100}$$

(10) $$H_{edge}=0{.7+25e}^{-h_{c}/58}$$

(11) $$Er_{center}{=48+401e}^{-h_{c}/98}$$

(12) $$H_{center}=0{.6+24e}^{-h_{c}/61}$$

The calculated *Er* and *H* are shown in Table 2. Based on the von Mises flow rule, Nix and Gao [37] used Tabor’s factor of 3 to convert the equivalent flow stress to hardness, *H*:

(13) $$H/H_{0}=\sqrt{1+h_{c}^{*}}/h_{c}$$

where *H*_0_ is the hardness that arises from the statistically stored dislocations alone and $h_{c}^{*}$ is a length that characterizes the depth dependence of the hardness. According to Equation (13), it is reasonable that hardness decreases with increasing contact depth. This is well consistent with the fact that the edge hardness is higher than that of the center.

### 3.6. Strengthening Mechanisms

Some studies have suggested that the comprehensive mechanical properties of materials prepared via ASR are distinctly better than those prepared by conventional rolling [12,14,38,39]. Typical strengthening methods of the UFG ferritic steel include grain refinement strengthening ($\Delta \sigma_{GB}$), solution strengthening ($\Delta \sigma_{SS}$), dislocation strengthening ($\Delta \sigma_{DS}$) and precipitation strengthening ($\Delta \sigma_{PS}$). Ignoring the interaction between each strengthening mechanism, the yield strength can be calculated by using the following equation:

(14) $$\sigma_{y}=\Delta \sigma_{PS}+\Delta \sigma_{DS}+\Delta \sigma_{SS}+\Delta \sigma_{GB}$$

Among them, because the carbon in ferrite is precipitated in the form of carbide, the content of C in ferrite is ignored [40]. In addition, the solubility of other alloying elements in ferrite of the medium-carbon low-alloy steel was simulated by JMatPro software. The results show that the solubility in ferrite is negligible, so $\Delta \sigma_{SS}$ is not considered in this study. 

#### 3.6.1. Dislocation Strengthening

As mentioned earlier, the ASR- and SR-steel have different grain sizes and dislocation densities. Moreover, the dislocation density of the ASR-steel is not uniform at the edge and in the center and the dislocation density at the edge is high while that is low in the center. This means that the structure gradient is formed. Thus, GNDs can better describe yield behavior according to Ramazani et al. [41]. As far as the dislocation strengthening is concerned, dislocations can be classified into two categories, i.e., statistically stored dislocations (SSDs), which do not create long-range lattice deformation (the sum of their Burgers Vector is zero) [42], and geometrically necessary dislocations (GNDs), which allow the accommodation of the lattice curvature. In general, GNDs are accumulated near the GBs and phase boundaries due to the lattice mismatch between neighboring grains, while SSDs are mostly stored in the interior of grains [8].

The differences in dislocation densities tested by EBSD (Figure 5) can help to explain why the ASR-steel has a higher strength than the SR-steel. The strength contribution of GNDs is calculated by using the following equation [43]:

(15) $$\sigma =M\alpha Gb\sqrt{\rho_{GNDs}}$$

where *α* is a constant related to material properties, which is generally 0.2–2.5 (here, α = 0.24 [44]), *M* is the orientation factor of 3.06, *G* is the shear modulus of ferrite with a value of 72 GPa and *b* is Burgers vector (0.248 nm).

Because of the fine grain sizes and nanosized precipitates in the ASR- and SR-steel, a large number of grain boundaries related to fine grains and nanosized particles are useful to store GNDs; consequently, GNDs significantly increase. Thus, dislocation strengthening is mainly contributed by the high density of GNDs, which has been proved in UFG ferritic steel during dynamic deformation [8]. Here, the strength increment of ASR-steel can be roughly estimated by GNDs. The maximum depth that was effectively refined equals approximately 100 μm per side of the steel, and this thickness is only 5% of the total thickness (100/2100 μm). Assuming the same dislocation density on both sides, the affected area at the edge is 10% while that in the center is 90%; thus, $\Delta \sigma_{DS}$ can be calculated by using the following equations:

(16) $$\sigma_{y}=0.1\times \sigma_{y-edge}+0.9\times \sigma_{y-center}$$

(17) $$\sigma_{DS-edge}=M\alpha Gb\sqrt{\rho_{GNDs-}{}_{edge}}$$

(18) $$\sigma_{DS-center}=M\alpha Gb\sqrt{\rho_{GNDs-center}}$$

The obtained results are listed in Table 3. We can see that $\Delta \sigma_{DS}$ of the two regions significantly decreases with increasing depth of the two steels. In particular, the $\Delta \sigma_{DS}$ values of ASR-steel are distinctly higher than those of the SR-steel. It can also be seen from Table 3 that the calculated values ($\sigma_{DS-total}$) of the two steels have the same trend as that indicated by the tensile tests (Figure 8). 

#### 3.6.2. Grain Boundary Strengthening

The contributions of the grain refinement to yield strength of the ASR- and SR steel can be calculated by using the following formula [45]:

(19) $$\Delta \sigma_{GB}=K_{y}d^{-1/2}$$

where *d* is the average effective grain size (the grain size *d* is calculated by referring to the values in Figure 4c) and *K_y_* is the influence coefficient of grain boundary on deformation. Generally, *K_y_* increases with the increase of solute carbon content; when the concentration of solute carbon increases continuously, the value of *K_y_* tends to be constant [46]. Here, *K_y_* is 455 MPa·μm^1/2^ for the medium-carbon low-alloy steel [9], thus, $\sigma_{GB}$ were obtained for the ASR- and SR-steel and summarized in Table 4. $\sigma_{GB}$ of the ASR-steel in two regions are significantly higher than those of the SR-steel; moreover, $\sigma_{GB}\;$ at the edge regions is distinctly higher than that in the central regions. In addition, by referring Equation (16), the calculated values ($\sigma_{GB-total})$ of the grain refinement strengthening in the ASR- and SR-steel are 431 MPa and 392 MPa, respectively.

#### 3.6.3. Precipitate Strengthening

**Figure 12:**
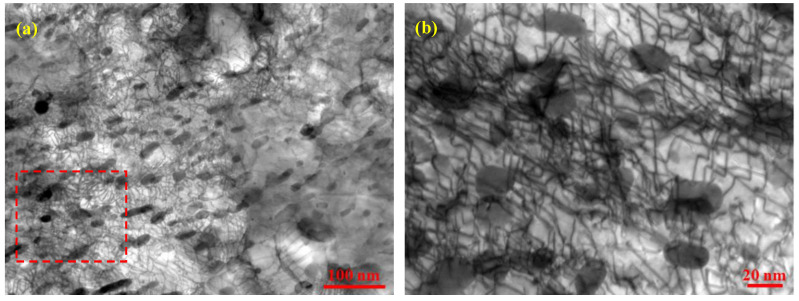
TEM pictures show the morphologies of the ASR-steel at the edge (**a**) and the magnified view of the region in the rectangle exhibits the Fe_3_C particles tangled with dense dislocations (**b**), obtained with g = 02$\bar{2}$.

As the TEM observations revealed, a large number of nanosized Fe_3_C particles dispersedly distribute in the ferritic matrixes of the ASR- and SR-steel (Figure 7). The postmortem TEM observations show that these Fe_3_C particles can be used as obstacles to dislocation slip (Figure 12). Similar to the morphology of the as-prepared steel, there are numerous nanosized particles in the matrix of the deformed ASR-steel (Figure 12a). However, the remarkable difference is that dense dislocations appear after the tensile deformation, and the majority of dislocations tangle with the particles. That is to say, the nanosized can effectively block the moving dislocations, thus leading to a significant increase in dislocation density around them. The close view reveals that dense dislocation nets form near the nanosized particles (Figure 12b); interestingly, some dislocations even cut through the particles, indicated by yellow arrows. This scene suggests that the nanosized Fe_3_C particles can not only contribute to the strength by blocking dislocations movement, but also retain ductility by allowing dislocations to slip through them. According to previous reports [6,7,47], the nanosized Fe_3_C precipitates can significantly increase the yield strength of steel.

As above mentioned, the average sizes of Fe_3_C at the edge and in the center of the ASR-steel are 24 nm and 22 nm while those are 32 nm and 37 nm for the SR-steel (Figure 7, Table 5), respectively. Meanwhile, for particles of the same size, the smaller the particle spacing, the higher the particle volume fraction, and the corresponding volume fraction of Fe_3_C is listed in Table 5. Thus, the contribution of precipitates to yield strength can be calculated by the Ashby—Orowan equation [48,49]:

(20) $$\sigma_{PS}=\frac{0.538Gbf^{1/2}}{\bar{d}}\ln\frac{\bar{d}}{2b}$$

where *σ_ps_* is the increment caused by precipitation strengthening, *b* is the Burgers vector (0.248 nm), *G* is the shear modulus (72 GPa for the Fe matrix), *f* and $\bar{d}$ are the volume fraction and the average size of Fe_3_C, respectively. Consequently, the *σ_ps_* values at various positions were obtained and summarized in Table 5. The results showed that the *σ_ps_* of the ASR-steel is 312 MPa, which is slightly higher than the value of 253 MPa for the SR-steel. 

**Figure 13:**
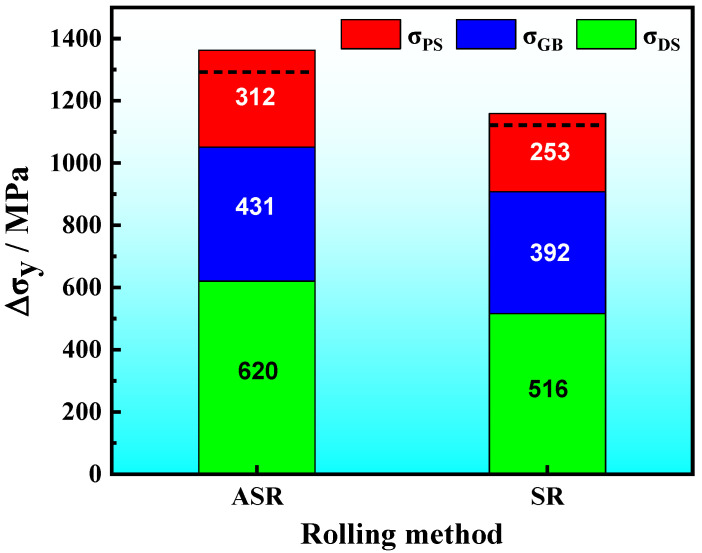
Schematic diagram showing the strengthening contributions caused by ASR and SR in the medium-carbon low-alloy steel.

The ultrahigh strength of the ASR-steel is the result of the joint actions of grain refinement strengthening, dislocation strengthening and precipitation strengthening. Figure 13 summarizes the roles of different strengthening mechanisms in the ASR- and SR-steel, where the actual values of *σ_y_* are indicated in black dotted lines. The differences between the theoretical calculation values and the tested results are 70 MPa and 36 MPa for the ASR- and SR-steel, respectively. The differences may be associated with the precision to measure the volume fraction of precipitates because TEM only provides two-dimensional images of the nanosized particles. Thus, the volume fraction of precipitates is inevitably overestimated, leading to the calculated values being higher than the results from tensile tests.

Among the strengthening mechanisms, it is noticed that dislocation strengthening is dominant while grain refinement strengthening is in second place. Moreover, the *σ_DS_* of the ASR-steel is significantly higher than that of the SR-steel. Based on EBSD, ASR-steel has the highest density of GNDs because the fine grains and nanosized precipitates contribute to numerous grain boundaries and interfaces (Figure 5 and Table 3); therefore, the contribution of dislocation strengthening to yield strength is the highest. At the same time, although the contribution of precipitation strengthening to yield strength is low compared to the other items, *σ_ps_* cannot be ignored. *σ_ps_* in the ASR-steel is as high as 312 MPa, which is six times as high as that of a Ce-containing low-carbon low-manganese TRIP steel, where *σ_ps_* is only 49 MPa [40]. This result suggests that it is possible to significantly increase the strength by increasing the volume fraction and decreasing the size of precipitates. Importantly, dislocation strengthening is extremely high in this study, especially for ASR-steel. This means that ASR is very effective in improving the material strength and the roles include not only refining grains but also increasing dislocation density.

## 4. Conclusions

In this study, a medium-carbon low-alloy steel was prepared via ASR in the austenitic region, compared to the compart prepared by SR. Subsequently, the effects of ASR and SR on the microstructure and mechanical properties of the prepared steels were explored, and the main conclusions can be drawn as follows:

(1) The microstructure of the ASR- and SR-steel is identical, consisting of ultrafine-grained ferrite and nanosized carbides. The average grain sizes of the ASR-steel at the edge and in the center are 850 nm and 1150 nm, respectively, which are significantly smaller than the values of 1300 nm and 1350 nm for the SR-steel. The key reason can be related to the large shear deformation at the edge during ASR, leading to effective grain refinement. 

(2) The yield strength and tensile strength of the ASR-steel are 1292 ± 10 MPa and 1357 ± 10 MPa, respectively, which are higher than the values of 1113 ± 10 MPa and 1185 ± 10 MPa for the SR-steel. Compared to the SR-steel, the ductility of the ASR-steel slightly decreases (~1%) while maintaining ultrahigh strength. The main reason is that the nanosized Fe_3_C particles can not only contribute to the strength by blocking dislocations movement, but also retain ductility by allowing dislocations to slip through them.

(3) The increase in yield strength of the ASR-steel is mainly related to the joint strengthening methods including grain refinement strengthening, dislocation strengthening and precipitation strengthening. Among them, dislocation strengthening and grain refinement strengthening are dominant, which are 620 MPa and 431 MPa, respectively. Despite the relative value of 311 MPa for the precipitate strengthening, the numerous nanosized Fe_3_C particles can effectively block dislocation movement, leading to the increasing dislocation density that is as high as 2.34 × 10^15^ m^−2^ at the edge of the ASR-steel.

(4) Nanoindentation shows that the *h_c_*, *Er* and *H* at the edge of the ASR-steel are higher than those of the center. Meanwhile, the pop-in phenomenon occurs during the loading-unloading tests, which is caused by the interaction between moving dislocations and obstacles such as the grain boundaries of the interfaces between nanosized precipitates and the matrix.

## Figures and Tables

**Table 1:** Chemical compositions of 45CrNiMoV steel (wt.%).

Element	C	Si	Ni	Cr	Mo	V	Mn	Al	Fe
Content	0.44	0.16	0.007	0.92	1.00	0.10	0.78	0.03	Bal

**Table 2:** Relationship among internal indentation of grain, contact depth (*h_c_*), elastic modulus (*Er*) and hardness (*H*) at the edge and in the center of the ASR-steel.

No.	*h_c_* ± 0.02 nm	*Er* ± 1 GPa	*H* ± 0.02 GPa
Edge-0	107	184	4.85
Edge-1	142	145	2.95
Edge-4	115	174	4.34
Edge-9	143	144	2.90
Center-0	150	137	2.64
Center-3	153	134	2.52
Center-5	149	135	2.65
Center-9	171	120	2.01

**Table 3:** Dislocation densities and the corresponding contributions to yield strength of the ASR- and SR-steel.

Region	$\rho_{GND}/m{-}^{2}$	$\sigma_{DS}/\mathrm{MPa}$	$\sigma_{DS-total}/\mathrm{MPa}$
ASR-steel-center	1.97 × 10^15^	614	620
ASR-steel-edge	2.34 × 10^15^	669	
SR-steel-center	1.38 × 10^15^	514	516
SR-steel-edge	1.49 × 10^15^	535	

**Table 4:** Grain size and the corresponding contributions to yield strength of the ASR- and SR-steel.

Region	Grain Size/μm	$\sigma_{GB}/\mathrm{MPa}$	$\sigma_{GB-total}/\mathrm{MPa}$
ASR-steel-center	1.15	424	431
ASR-steel-edge	0.85	494	
SR-steel-center	1.35	392	392
SR-steel-edge	1.30	399	

**Table 5:** Average size and volume fraction of Fe_3_C together with the corresponding contribution to yield strength in the ASR- and SR-steel.

Region	Average Size of Fe_3_C/±1 nm	Volume Fraction/%	$\sigma_{PS}/\mathrm{MPa}$	$\sigma_{PS-total}/\mathrm{MPa}$
ASR-steel-center	24	4.1	315	312
ASR-steel-edge	22	3.5	305	
SR-steel-center	37	4.9	249	253
SR-steel-edge	32	4.5	268	

## Data Availability

All data included in this study are available upon request by contact with the corresponding author.
